# N-Dimensional LLL Reduction Algorithm with Pivoted Reflection

**DOI:** 10.3390/s18010283

**Published:** 2018-01-19

**Authors:** Zhongliang Deng, Di Zhu, Lu Yin

**Affiliations:** School of Electronic Engineering, Beijing University of Posts and Telecommunications, No. 10 Xitucheng Road, Beijing 100876, China; dengzhl@bupt.edu.cn (Z.D.); inlu_mail@163.com (L.Y.)

**Keywords:** LLL reduction, pivoted reflection, integer least squares (ILS), global navigation satellite system (GNSS)

## Abstract

The Lenstra-Lenstra-Lovász (LLL) lattice reduction algorithm and many of its variants have been widely used by cryptography, multiple-input-multiple-output (MIMO) communication systems and carrier phase positioning in global navigation satellite system (GNSS) to solve the integer least squares (ILS) problem. In this paper, we propose an n-dimensional LLL reduction algorithm (*n*-LLL), expanding the Lovász condition in LLL algorithm to n-dimensional space in order to obtain a further reduced basis. We also introduce pivoted Householder reflection into the algorithm to optimize the reduction time. For an *m*-order positive definite matrix, analysis shows that the *n*-LLL reduction algorithm will converge within finite steps and always produce better results than the original LLL reduction algorithm with *n* > 2. The simulations clearly prove that *n*-LLL is better than the original LLL in reducing the condition number of an ill-conditioned input matrix with 39% improvement on average for typical cases, which can significantly reduce the searching space for solving ILS problem. The simulation results also show that the pivoted reflection has significantly declined the number of swaps in the algorithm by 57%, making *n*-LLL a more practical reduction algorithm.

## 1. Introduction

With the rapid development of the Beidou System (BDS), the Galileo system, the Global Positioning System (GPS) and the GLONASS system, the Global Navigation Satellite System (GNSS) is serving more and more people with higher positioning accuracy [1,2]. Alongside the standard point positioning, carrier-phase based precise positioning techniques with cm-level to mm-level accuracy, such like real-time kinematic (RTK) and precise point positioning (PPP), have started to show their potential in public services other than in specific areas such as ground surveying. The commercial continuous operational reference station (CORS) network providers have enabled the precise positioning applications in unmanned aerial vehicle (UAV), unmanned autonomous vehicle and so on [3,4,5].

The main computational effort in carrier-phase precise positioning is to resolve the carrier phase integer ambiguity which is contaminated by all kind of noises during the signal propagation. Several efficient ambiguity resolution methods were proposed during the last several decades such as: Least-Square Ambiguity Searching Technique (LSAST) [6], Triple Frequency Ambiguity Resolution (TCAR) [7], Least-squares AMBiguity Decorrelation Adjustment (LAMBDA) [8,9]. The great breakthrough of LAMBDA algorithm is bringing the “decorrelation” process into integer ambiguity resolution, dividing the whole process into: estimation, decorrelation (also known as Z-transformation), search and back transformation.

As the measurements of pseudo-range and carrier phase are strongly correlated in time and space, the coefficient matrix to resolve the ambiguities is so ill-conditioned that the search space is huge and abnormal, making the search process extremely time-consuming and inefficient. For real-time applications, the decorrelation process is curial to reduce search effort. As for LAMBDA, integer Gauss transformation with permutation is used as the decorrelation process and is proven to be very effective. A series of algorithms have been applied to the decorrelation process since then. Xu used Cholesky decomposition to calculate the Z-transformation matrix [10]; Chang modified the Gauss transformation in LAMBDA with symmetric pivoting strategy [11]; Xu proposed the parallel Cholesky-based reduction method using minimum pivoting strategy [12] and Hassibi was the first to introduce LLL algorithm into integer ambiguity resolution [13].

The LLL reduction algorithm was first proposed by Arjen Lenstra, Hendrik Lenstra and László Lovász in 1982 [14] and had been proven useful polynomial-time algorithm to solve the closest vector problem (CVP) since then. With the development of lattice theory and its application, LLL algorithm becomes a powerful tool to solve the ILS problem, which expands its usage to numerous applications such like next generation MIMO communication detection algorithm [15,16,17], integer ambiguity resolution [13,18] and many other integer solution finding problem.

The original LLL reduction algorithm uses Gram-Schmidt orthogonalization to generate orthogonal basis, which involves O(*n*log(B))-bit integer. A float point LLL (FPLLL) algorithm is proposed to avoid the waste of resources [19]. Schnorr used the half-k method [20] to ensure the calculation accuracy with FPLLL, which only involves O(*n* + log(B))-bit integer and converges in O(*n*^4^log(B)). Koy proposed a segment LLL algorithm, weakening the constraints of reduction to ensure the efficiency of the algorithm when dealing with matrix of rank 350 or above. Schnoor proposed the deep insertion LLL (DeepLLL) and the Block Korkine-Zolotareff (BKZ) algorithm [21], which improved the performance significantly. Fontein made the DeepLLL algorithm a polynomial-time algorithm with the help of potential factor, naming it potential LLL (PotLLL) [22].

In this paper, we propose the *n*-LLL reduction, expanding the Lovász condition of original LLL algorithm to n-dimensional space. And we give out an adjustable parameter “*n*” to balance the performance and computational efforts. Pivoted Householder reflection and Givens transformation are also introduced into the *n*-LLL algorithm to further optimize the reduction time.

The performance and complexity of the *n*-LLL algorithm are then analyzed, showing that the new algorithm performs better than the original LLL algorithm with *n* > 2 and will always converge in polynomial time. The simulation results show that the *n*-LLL algorithm has better reduction quality than the original LLL with about 39% improvement on average. The new algorithm causes no significant increase in computational efforts because of the pivoted reflection, which is able to reduce as much as 57% swaps in the algorithm.

## 2. The LLL Reduction

A lattice is defined as:

(1) $$L=\left\{\sum_{i=1}^{m}a_{i}b_{i}|a_{i}\in \mathbb{Z}\right\}$$

where $b_{i}$ denote m independent liner vectors defined on ${\mathbb{R}}^{n}$ and are called a basis of lattice L. So, a lattice can be seen as a discrete point set inside the real value space ${\mathbb{R}}^{n}$.

Then the typical weighted ILS problem like:

(2) $$\underset{a\in {\mathbb{Z}}^{n}}{\min}{\left\|\hat{a}-a\right\|}_{Q_{\hat{a}}^{-1}}^{2}$$

can be described as: finding a vector a∈L that is closest to $\hat{a}\in {\mathbb{R}}^{n}$ ($Q_{\hat{a}}$ is the covariance matrix of vector $\hat{a}$), which is also known as a CVP. As a CVP is an NP-hard problem, one efficient approach to acquire an approximate solution is lattice basis reduction. To simplify the demonstration, the discussions below involve only square matrix.

For a basis $B=\left(b_{1},b_{2},b_{3}\cdots b_{m}\right)$ of lattice L, obviously $B_{o}=B\cdot T$ is also a basis of L, where T is a unimodular matrix. Then the weighted ILS problem (2) can be rewritten as:

(3) $$\underset{z\in {\mathbb{Z}}^{n}}{\min}{\left\|\hat{z}-z\right\|}_{Q_{\hat{z}}^{-1}}^{2}$$

where:

(4) $$\begin{array}{l} \hat{z}=T^{-1}\cdot \hat{a}\\ z=T^{-1}\cdot a\\ Q_{\hat{z}}^{-1}=T^{T}\cdot Q_{\hat{a}}^{-1}\cdot T \end{array}$$

Assuming that we are able to find a proper unimodular matrix T that makes all the column vectors of $B_{o}$ mutually orthogonal, the optimized solution of z can be obtained by rounding each entry in $\hat{z}$ and the solution for a can be calculated accordingly, which is also known as the Babai’s method [23]. However, such kind of matrix T cannot be found in general cases. As a result, the best way we can do is to find an approximate solution for matrix T that makes $B_{o}$ as orthogonal as possible and the column vectors as short as possible, which is the so called “reduction” process.

**Figure 1:**
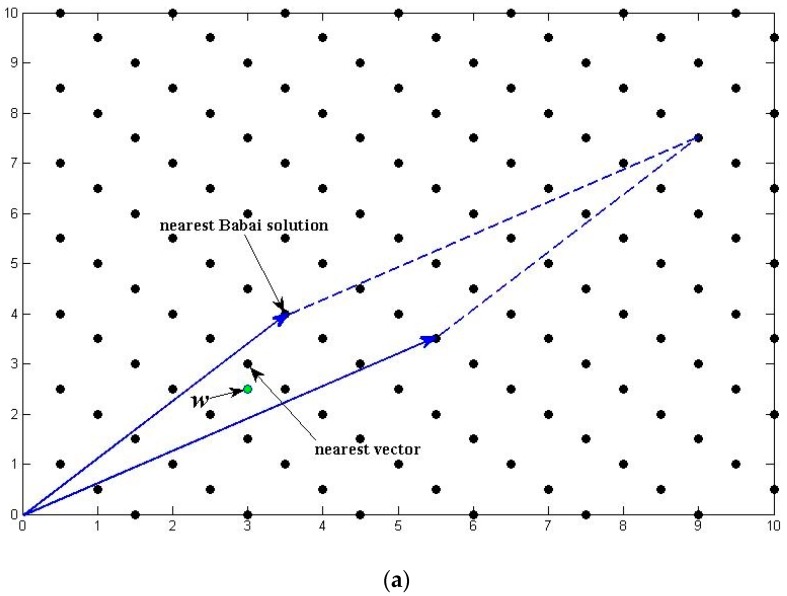

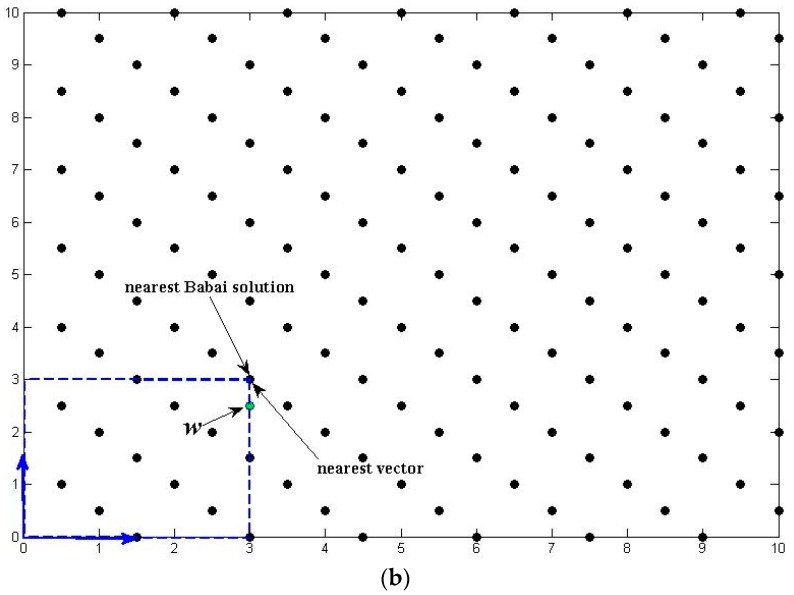
Illustration of “Bad” and “Good” lattice basis.

Figure 1 shows the difference between a “bad” and a “good” reduced lattice basis and how they affect the search process. The basis vectors in Figure 1a are relatively long and have smaller angle, which will bring larger error when estimating the CVP solution for real value vector w. On the contrary, the basis vectors in Figure 1b are mutually orthogonal, enabling the Babai’s method to find the solution instantly.

### 2.1. The LLL Reduction Algorithm

The two primary goals of reduction process are:

- To make the column vectors in $B_{o}$ as mutually orthogonal as possible. As mentioned above, if the vectors are mutually orthogonal, then a simple rounding process will solve the ILS problem. Thus, the orthogonality of the vectors actually defines shape of search space, which will have significant influence on search efficiency;
- To minimize the length of vectors $b_{i}$. Note that $\prod_{i=1}^{m}\left\|b_{i}\right\|$ gives an upper bound of the volume of searching space, which means minimizing the vector length will shrinking the search space.

With the two goals, several reduction algorithm were proposed in the last decades and among those was the most famous LLL reduction algorithm proposed by Lenstra et al. [14].

The LLL reduction algorithm is consisted of two parts: size reduction and vector swap. The size reduction uses Gram-Schmidt orthogonalization. Let $B=\left(b_{1},b_{2},b_{3}\cdots b_{m}\right)$ be a basis of lattice L and a Gram-Schmidt basis $B^{*}$ can be described as:

(5) $$b_{i}^{*}=b_{i}-\sum_{j=1}^{i-1}\mu_{i,j}b_{j}^{*}$$

where:

(6) $$\mu_{i,j}=\frac{\langle b_{i},b_{j}^{*}\rangle }{\langle b_{j}^{*},b_{j}^{*}\rangle }$$

**Definition** **1.** *Given a basis $B\in {\mathbb{R}}^{m\times m}$ of lattice L and its Gram-Schmidt orthogonal matrix $B^{*}$, B is LLL reduced if it satisfies the following two conditions:*

(7) $$|\mu_{i,j}|\leq \frac{1}{2}\;\mathrm{for}\;1\leq j<i\leq m$$

(8) $$\delta {\left\|b_{i-1}^{*}\right\|}^{2}\leq {\left\|b_{i}^{*}+\mu_{i,i-1}b_{i-1}^{*}\right\|}^{2}\;\mathrm{for}\;1<i\leq m$$

*where parameter δ satisfies $\frac{1}{4}<\delta <1$.*

The LLL reduction algorithm starts with setting $b_{1}^{*}=b_{1}$ and then $b_{i}$ is replaced by $b_{i}-{\left[\mu_{i,j}\right]}_{round}b_{j}^{}$ if $\left|\mu_{i,j}\right|\leq \frac{1}{2}$ for 1≤j<i≤m to satisfy the size condition (7). If Lovász condition (8) is violated for 1<i≤m, then the column vectors $b_{i-1}$ and $b_{i}$ are swapped and the size reduction process will go back to $b_{i-1}$ again. After as much as $O\left(n^{2}\log B\right)$ iterations [14], when the Lovász condition is satisfied between $b_{m-1}$ and $b_{m}$, the LLL reduction is done.

As we can see from the process of LLL reduction algorithm shown in Algorithm 1, the size reduction process adjusts the angle of $b_{i}$ by rounded Gram-Schmidt orthogonalization and the length of $b_{i}$ is reduced at the same time.
**Algorithm 1:** The LLL reduction algorithm**Set**
$b_{1}^{*}=b_{1}$**Set**
*i* = 2**For**
i≤m **For** j = 1 **to**
*i* − 1  **Set**
$b_{i}$ = $b_{i}-{\left[\mu_{i,j}\right]}_{round}b_{j}^{}$         (Size reduction) **End** **If**
$\delta {\left\|b_{i-1}^{*}\right\|}^{2}>{\left\|b_{i}^{*}+\mu_{i-1,i}b_{i}^{*}\right\|}^{2}$
**then**   (Lovász condition)  **Swap** ($b_{i-1}$, $b_{i}$)              (Swap process)  **Set**
*i* = max(*i* − 1, 2) **Else**  Set *i* = *i* + 1  **End****End**

Clearly, the performance of size reduction process is strongly dependent on the order of the column vectors. Putting shorter vectors ahead will help improving the performance because shorter vectors kind of “push” the vectors after them to a more orthogonal angle in order to satisfy the size condition. Ideally, if we have:

(9) $$\left\|b_{1}\right\|\leq \left\|b_{2}\right\|\leq \left\|b_{3}\right\|\cdots \leq \left\|b_{m}\right\|$$

and then the lattice can be most reduced. But unfortunately, there is still no algorithm can achieve (9) in polynomial time. As a result, Lovász set the condition to (8), where δ is usually set to 0.75 [14], in order to make the algorithm finish within polynomial time.

### 2.2. The LLL Reduction with Pivoted Reflection

As can be seen from Algorithm 1, the loop indicator *i* decreases only when the swaps take place, which means the number of iterations is highly dependent on the number of swaps. Hence, if the column vectors $b_{i}$ are in ascending order or at least as ascending ordered as possible before the LLL reduction is executed, the number of swaps as well as the reduction time will surely decline. 

Motivated by Chang’s MLAMBDA [11] which utilizes the symmetric pivoting strategy to improve the efficiency of the reduction process and Wübben’s MMSE Sorted QR decomposition [24] which extends the V-BLAST algorithm, we introduce the pivoted reflection strategy into LLL algorithm to pre-sort the column vectors.

As the Gram-Schmidt orthogonalization process in the original LLL algorithm is not an isometric process, the pivoting of the vectors has limited influence on the reduction time. Therefore, we chose the Householder transformation which is an elementary reflection transformation proposed by Turnbull and Aitken in 1932. The typical usage of the Householder reflection is QR decomposition. Given a none-zero vector $b\in {\mathbb{R}}^{n}$, one can construct vector u=b+ρe with $e={\left[1,\underset{n-1zeros}{\underset{︸}{0\cdots 0}}\right]}^{T}$ and $\rho =sign(b_{1})\cdot \left\|b\right\|$. Then the transformation matrix can be constructed as:

(10) $$H=I-2\frac{u\cdot u^{T}}{{\left\|u\right\|}^{2}}$$

Assuming *i* − 1 vectors of B have been transformed and $B_{i}$ is the submatrix:

(11) $$B_{i}=\left[\begin{array}{ccc} b_{i,i} & \cdots & b_{1,m}\\ \vdots & \ddots & \vdots \\ b_{m,1} & \cdots & b_{m,m} \end{array}\right]$$

then the *i*th transformation matrix can be calculated as:

(12) $$H_{i}=\left[\begin{array}{cc} I_{i-1} & 0\\ 0 & H_{B_{i}} \end{array}\right]$$

where $H_{B_{i}}$ is the transformation matrix for submatrix $B_{i}$ according to (10). Then we get:

(13) $$R=H_{m-1}H_{m-2}\cdots H_{1}B\;\mathrm{and}\;Q=H_{1}H_{2}\cdots H_{m-1}$$

with B=Q·R, where Q is an orthogonal matrix and R is an upper triangle matrix. 

In order to make the column vectors as ascending ordered as possible, before each Householder transformation the shortest vector in submatrix $B_{i}=\left[b_{1}^{i},b_{2}^{i},b_{3}^{i}\cdots b_{m-i+1}^{i}\right]$ is moved ahead and the corresponding column vector in B is swapped with $b_{i}$ accordingly. The whole LLL reduction with pivoted reflection is given in Algorithm 2.
**Algorithm 2:** The LLL reduction with pivoted reflection**Set**
R=B and $Q=I_{m\times m}$**For**
*i* = 1 to m − 1         (Pivoted Householder Reflection) **Find** the shortest vector $b_{s}^{i}$ in $B_{i}$ **Swap**($b_{i}$, $b_{i+s-1}$) of B **Calculate**
$H_{i}$ for B **Set**
$R=H_{i}\cdot R$ and $Q=Q\cdot H_{i}$**End****Set**
*k* = 2**While**
k≤m **For** j = *k*-1 **down to** 1 **Set**
$r_{k}$ = $r_{k}-{\left[\frac{r_{j,k}}{r_{j,j}}\right]}_{round}\cdot r_{j}$          (Size reduction) **End** **If**
$\delta \cdot r_{k-1,k-1}^{2}>r_{k,k}^{2}+r_{k-1,k}^{2}$
**then**      (Lovász condition)  **Swap** ($r_{k-1}$,$r_{k}$)               (Swap process)  **Calculate**
$H_{k-1}$ for R  **Set**
$R=H_{k-1}\cdot R$ and $Q=Q\cdot H_{k-1}$ **Set**
*k* = max(*k* − 1, 2) **Else**  Set *k* = *k* + 1  **End****End**

Furthermore, the algorithm can be more efficient by applying Givens transformation to the rotations in the swap process instead of Householder reflection, because only $b_{i,i-1}$ needs to be transformed to zero when swapping $b_{i-1}$ and $b_{i}$. It should also be emphasized that the pivoted reflection strategy does not always sort the vectors in perfect ascending order, because the reflection on $b_{i}$ also transforms the vectors after it, which may create shorter vector. But as can be seen in the simulations in Section 4, the pivoted reflection is proved to be very effective in minimizing the number of vector swaps in LLL algorithm in many cases of interest.

## 3. N-Dimensional Expansion of LLL Reduction

As discussed at the end of Section 2.1, the reduction quality and the improvement of search space are both highly dependent on the order of the basis vectors. We propose the *n*-LLL reduction algorithm, which inherits the basic outline of the LLL reduction algorithm and strengths the constraint of the order of basis vectors. 

### 3.1. The N-Dimensional LLL Reduction Algorithm

Look back at the two conditions of LLL reduction again:

$$|\mu_{i,j}|\leq \frac{1}{2},\;\delta {\left\|b_{i-1}^{*}\right\|}^{2}\leq {\left\|b_{i}^{*}+\mu_{i,i-1}b_{i-1}^{*}\right\|}^{2}$$

and the reduction process can be described in another way as: applying a series of Gaussian reductions in the 2-dimensional lattice spanned by a Lovász condition optimized vector pair $b_{i-1}$ and $b_{i}$.

However, the Lovász condition here only focuses on local optimization within two neighbor vectors which ignores the global optimization. In order to improve the effect of the optimization to enhance the ordering constraint, we extend the 2-dimensional condition to *n*-dimension. Like the LLL reduction defined in Definition 1, the n-dimensional LLL reduction is defined as:

**Definition** **2.** *Given a basis $B\in {\mathbb{R}}^{m\times m}$ of Lattice L and its Gram-Schmidt orthogonal matrix $B^{*}$, B is n-LLL reduced (2≤n<m) if it satisfy the following two conditions:*

(14) $$|\mu_{i,j}|\leq \frac{1}{2}\;\mathrm{for}\;1\leq j<i\leq m$$

(15) $$\delta {\left\|b_{i-1}^{*}\right\|}^{2}\leq \min\left({\left\|b_{i}^{*}+\mu_{i,i-1}b_{i-1}^{*}\right\|}^{2},\cdots ,{\left\|b_{i+n-2}^{*}+\sum_{j=i-1}^{\min(i+n-3,m)}\mu_{i+n-2,j}b_{j}^{*}\right\|}^{2}\right)\;\mathrm{for}\;1<i\leq m$$

*where parameter δ satisfies: $\frac{1}{4}<\delta <1$.*

Condition (15) can also be rewritten as:

(16) $$\delta {\left\|b_{i-1}^{*}\right\|}^{2}\leq \lambda_{1}{\left(\left[b_{1}^{i},b_{2}^{i},b_{3}^{i}\cdots b_{\min(n-1,m-i+1)}^{i}\right]\right)}^{2}$$

where $b_{k}^{i}$ is the column vector in submatrix $B_{i}$ and $\lambda_{1}\left(B\right)$ implies the length of the shortest none-zero vector in B. 

Both condition (15) and (16) ensure that $b_{i-1}^{}$ is the optimized choice in the following n vectors, which brings stronger constraint to the reduced basis than the original Lovász condition. The *n*-LLL reduction becomes LLL reduction when *n* = 2.

Note that the extend Lovász condition in the *n*-LLL reduction is similar to Block Korkine-Zolotarev (BKZ) [21] reduction that applies Korkine-Zolotarev (KZ) reduction within a k-block. This paper extends the original Lovász condition instead of introducing KZ reduction basis.

As the constraint in *n*-LLL is stronger than in the original one, the increase of reduction time is predictable. Thus, pivoted reflection mentioned in Section 2.2 plays a vital role in controlling the total reduction time of the algorithm. So, we fuse QR decomposition and reduction together by deeply coupling the pivoted reflection and *n*-LLL reduction process.

One possible algorithm to achieve *n*-LLL reduction is shown in Algorithm 3:
**Algorithm 3:** The *n*-LLL reduction with pivoted reflection**Set**
R=B and $Q=I_{m\times m}$(Move the shortest vector to $r_{1}$)**Find** the shortest vector $r_{s}$ in R**Swap**($r_{i}$,$r_{s}$) of R**Calculate**
$H_{1}$ for R**Set**
$R=H_{1}\cdot R$ and $Q=Q\cdot H_{1}$**Set**
*i* = 2**While**
i≤m           (Pivoted reflection and reduction process) **Find** the shortest vector $r_{s}^{i}$ in $R_{i}$ **Swap**($r_{i}$, $r_{i+s-1}$) of R **Set** temp = *i* **For**
*j* = *i* − 1 **down to** 1  **Set**
$r_{k}$ = $r_{i}-{\left[\frac{r_{j,i}}{r_{j,j}}\right]}_{round}\cdot r_{j}$                (Size reduction)  **If**
$\delta \cdot r_{j,j}^{2}>{\left\|b_{temp-j+1}^{j}\right\|}^{2}$
**and**
*i* − *j* < *n*
**then**   (Extended Lovász condition)   **Swap** ($r_{j}$, $r_{temp}$)                   (Swap process)   **Set** temp = *j*  **End** **End** **Calculate**
$H_{temp}$ for R **Set**
$R=H_{temp}\cdot R$ and $Q=Q\cdot H_{temp}$ **If**
*i* ! = temp **then**  *i* = temp **Else**  **Set**
*i* = *i* + 1 **End****End**

The algorithm above uses the “Pivoting-Reduction-Reflection” strategy for each vector of B instead of “Pivoting-Reflection and Reduction” strategy of Algorithm 2. The pivoting process in the fusion strategy gives better result as it takes the influence of the rotations in vector swap into consideration. Note that vector $r_{i}$ may have less than m−i zero entries because the vector pivoted to $r_{i}$ has not been reflected yet. However, as the Householder reflection is an isometry, it will not be a problem in the swap process. The vector is then reflected after swapped to a proper place. The whole algorithm is actually executing the pivoted QR decomposition and *n*-LLL reduction in parallel, making it more efficient.

### 3.2. Analysis

#### 3.2.1. The Performance

The *n*-LLL reduction algorithm strengthens the Lovász condition, which will certainly improve the quality of reduced basis. In this section, the performance improvement between the *n*-LLL and LLL reduction is detailed analyzed.

In order to compare the performance of reduction algorithm, we need to introduce measures to evaluate the reduction quality. As the size reduction processes of the two algorithms are the same, we only need to compare the orthogonality of the reduced basis according to the two goals mentioned in Section 2. One of the mostly used way to measure the orthogonality defects is imported from the Hadamard inequality:

**Theorem** **1.** *(Hadamard Inequality): Given a lattice L and one of its basis $B\in {\mathbb{R}}^{m\times m}$, we have:*

(17) $$\det L=\det\left(B\right)=\prod_{i=1}^{m}\left\|b_{i}^{*}\right\|\leq \prod_{i=1}^{m}\left\|b_{i}\right\|$$

*and it becomes an equation if and only if all the vectors are mutually orthogonal. Then, the orthogonality defect factor can be defined as:*

(18) $$OD\left(L\left(B\right)\right)=\frac{\prod_{i=1}^{m}\left\|b_{i}\right\|}{\det L}$$

*with 1≤O(L(B)) [25].*

According to (15), we have:

(19) $$\delta {\left\|b_{i-1}^{*}\right\|}^{2}\leq {\left\|b_{i}^{*}\right\|}^{2}+\mu_{i,i-1}^{2}{\left\|b_{i-1}^{*}\right\|}^{2}$$

(20) $$\delta {\left\|b_{i-1}^{*}\right\|}^{2}\leq {\left\|b_{i+1}^{*}\right\|}^{2}+\mu_{i+1,i}^{2}{\left\|b_{i}^{*}\right\|}^{2}+\mu_{i+1,i-1}^{2}{\left\|b_{i-1}^{*}\right\|}^{2}$$

······

(21) $$\delta {\left\|b_{i-1}^{*}\right\|}^{2}\leq {\left\|b_{i+n-2}^{*}\right\|}^{2}+\sum_{j=i-1}^{\min(i+n-3,m)}\mu_{i+n-2,j}^{2}{\left\|b_{j}^{*}\right\|}^{2}$$

and the size reduction process makes sure that $\left|\mu_{i,j}\right|\leq \frac{1}{2}$. As a result, (19) can be rewritten as:

(22) $${\left\|b_{i}^{*}\right\|}^{2}\geq \left(\delta -\frac{1}{4}\right){\left\|b_{i-1}^{*}\right\|}^{2}$$

which means:

(23) $${\left\|b_{i+1}^{*}\right\|}^{2}\geq \left(\delta -\frac{1}{4}\right){\left\|b_{i}^{*}\right\|}^{2}$$

By combining (23) and (20), we can get:

(24) $${\left\|b_{i+1}^{*}\right\|}^{2}\geq \frac{{\left(\delta -\frac{1}{4}\right)}^{2}{\left\|b_{i-1}^{*}\right\|}^{2}}{\delta }$$

Repeatedly:

(25) $$\left\{ \begin{array}{l} {\left\|b_{j}^{*}\right\|}^{2}\leq k^{i-j}{\left\|b_{i}^{*}\right\|}^{2}\\ k=\frac{\delta \cdot \delta^{\frac{1}{1-n}}}{\left(\delta -\frac{1}{4}\right)} \end{array}\right.,\frac{i-j}{n-1}\in \mathbb{Z}$$

Furthermore, by substituting (25) into the Gram-Schmidt orthogonalization (5), we get:

(26) ‖bi‖2=‖bi∗‖2+∑j=1i−1μi,j2‖bj∗‖2≤‖bi∗‖2+14∑j=1i−1‖bj∗‖2≤34‖bi∗‖2+‖bi∗‖24∑j=0⌊i−1n−1⌋kn(n−1)j∑l=1n−1kll−1≤34‖bi∗‖2+‖bi∗‖24∑j=0in−1−1kn(n−1)jkn−1δk2−1=34‖bi∗‖2+‖bi∗‖24kni−1knn−1−1kn−1δk2−1

where ⌊a⌋ denotes the integer no larger than a. By considering $1<\delta k_{2}<k_{n}\leq k_{2}\leq k_{n}^{n-1}$, (26) can be further rewritten to:

(27) $${\left\|b_{i}\right\|}^{2}\leq \frac{k_{n}^{i}}{\delta k_{2}-1}{\left\|b_{i}^{*}\right\|}^{2}$$

Then the orthogonality defect factor is calculated:

(28) OD(L)=∏i=1m‖bi‖detL≤∏i=1mkniδk2−1‖bi∗‖2∏i=1m‖bi∗‖2=knm(m+1)4(δk2−1)m2

It can be seen clearly from (28) and (25) that the upper bound of the orthogonality defect factor declines as the parameter n increasing, which proves that the reduction quality of *n*-LLL reduction algorithm with *n* > 2 is better than the original LLL reduction (which is equivalent to 2-LLL).

It should be emphasized that the orthogonality does not directly affect the search time according to [26,27]. However, as the two algorithms compared through the orthogonality defect factor share the same size reduction constraint, the difference of the factor actually measures the performance of vector ordering, or in another way measures the efficiency of size reduction. So, the conclusion of (28) still holds in numerical simulations in Section 4.

#### 3.2.2. The Complexity

As can be seen in Algorithm 3, the loop indicator *i* goes back to *temp* after each vector swap, where *temp* indicates the final position that $b_{i}$ is placed. Therefore, with *temp* < *i*, it is hard to determine that whether the algorithm will converge. Here we give the proof that the *n*-LLL reduction algorithm will converge in polynomial time. 

Let:

(29) $$L_{i}=\left\{\sum_{k=1}^{i}a_{k}b_{k}|a_{k}\in \mathbb{Z}\right\}$$

be a sub-lattice of lattice L and we define:

(30) $$d_{i}=\prod_{k=1}^{i}{\left\|b_{k}\right\|}^{2}$$

(31) $$D_{i}=\prod_{i=1}^{m}d_{i}$$

where we have $\det{\left(L_{i}\right)}^{2}=d_{i}$.

In the *n*-LLL algorithm, the value of $D_{i}$ changes only when the vector swap is executed. To be more accurate, only the elements from $d_{\alpha }$ to $d_{\beta -1}$ in $D_{i}$ will change when swapping $b_{\alpha }$ and $b_{\beta }$. And the swap happens only when the extended Lovász condition is violated, which means:

(32) $${\left\|b_{\beta }^{*}\right\|}^{2}<\delta {\left\|b_{\alpha }^{*}\right\|}^{2}-\sum_{j=\alpha }^{\beta -1}{\left\|\mu_{\beta ,j}b_{j}^{*}\right\|}^{2}\leq \delta {\left\|b_{\alpha }^{*}\right\|}^{2}$$

Therefore, the old $d_{\varepsilon }^{old}$ will change to $d_{\varepsilon }^{new}$ (α≤ε<β):

(33) $$\begin{array}{ll} d_{\varepsilon }^{new} & ={\left\|b_{1}^{*}\right\|}^{2}\cdot {\left\|b_{2}^{*}\right\|}^{2}\cdots {\left\|b_{\beta }^{*}\right\|}^{2}\cdots {\left\|b_{\varepsilon }^{*}\right\|}^{2}\\ & ={\left\|b_{1}^{*}\right\|}^{2}\cdot {\left\|b_{2}^{*}\right\|}^{2}\cdots {\left\|b_{\alpha }^{*}\right\|}^{2}\cdots {\left\|b_{\varepsilon }^{*}\right\|}^{2}\cdot \frac{{\left\|b_{\beta }^{*}\right\|}^{2}}{{\left\|b_{\alpha }^{*}\right\|}^{2}}\\ & \leq d_{\varepsilon }^{old}\cdot \delta \end{array}$$

and the maximum change of $D_{i}$ by one swap is $\delta^{n-1}$.

According to the Hermite principle, for a lattice $L\subset {\mathbb{Z}}^{m}$ we can have:

(34) $$\underset{0\neq v\in L_{i}}{\min}\left\|v\right\|\leq \sqrt{i}\cdot \det{\left(L_{i}\right)}^{2}$$

which also means:

(35) $$D_{m}=\prod_{i=1}^{m}d_{i}=\prod_{i=1}^{m}\det{\left(L_{i}\right)}^{2}\geq \prod_{i=1}^{m}i^{-i}={\left(m!\right)}^{-m}\geq m^{-m^{2}}$$

As a result, the value of $D_{m}$ has a certain lower bound, which means only finite number of swaps are executed during the whole algorithm. Assuming that $D_{m}=D_{start}$ at the beginning of the algorithm and $D_{m}=D_{end}$ at the end, we have:

(36) $$m^{-m^{2}}\leq D_{end}\leq {\left(\delta^{n-1}\right)}^{N}D_{start}$$

where ***N*** denotes the total number of vector swaps. Considering that δ<1, (36) can be rewritten as:

(37) $$N=\frac{O\left(m^{2}\log m+\log D_{end}\right)}{n-1}$$

and we also have:

(38) $$D_{end}=\prod_{i=1}^{m}{\left\|b_{i}^{*}\right\|}^{2(m+1-i)}\leq \prod_{i=1}^{m}{\left\|b_{i}\right\|}^{2(m+1-i)}\leq {\left(\max\left\|b_{i}\right\|\right)}^{m(m+1)}=B^{m(m+1)}$$

where *B* denotes the longest vector in B.

Therefore, the total number swaps in the algorithm is:

(39) $$N=O\left(n^{-1}\cdot m^{2}\log B\right)$$

The fact is that the number of swaps declines as parameter *n* increases. However, the actual calculation effort is related to the number of loops. As the loop indicator *i* goes back as much as *n* steps after each vector swap instead of 1 step in the original LLL reduction algorithm which makes the maximum number of loops n·N+m, which means each swap takes O(n·logn) basic steps to satisfy extended Lovász condition in the n dimensional lattice space. Thus, $O\left(\log n\cdot m^{3}\log B\right)$ basic steps are needed just to check all the vectors. Obviously, given an m rank basis, the calculation effort increases along with the increase of parameter *n* and the total reduction time remains polynomial for all n≥2.

## 4. Experiments and Results

### 4.1. Measures of Reduction Quality

In Section 3.2, we introduced the orthogonality defect factor to compare the *n*-LLL and the original LLL reduction algorithm. And we also mentioned that the orthogonality defect factor only measures the orthogonal quality of the two algorithms. In this section, we introduce a more practical measures, which is easier to calculate through the reduced matrix: the condition number [10].

The searching region which contains the nearest solution of ILS problem (3) can be written as:

(40) $${\left(\hat{z}-z\right)}^{T}Q_{\hat{z}}^{-1}\left(\hat{z}-z\right)<\chi^{2}$$

and shape of this hyper-ellipsoid is determined by the ratio of the major and minor axes, which can be described as:

(41) $$elongation=\sqrt{\frac{\lambda_{\max}}{\lambda_{\min}}}=\sqrt{\kappa \left(Q_{\hat{z}}^{}\right)}$$

where $\kappa \left(Q_{\hat{z}}^{}\right)$ is defined as the condition number of $Q_{\hat{z}}^{}$, $\lambda_{\max}$ and $\lambda_{\min}$ are the maximum and minimum eigenvalues of $Q_{\hat{z}}^{}$.

As a matter of fact, the condition number measures the elongation of the searching hyper-ellipsoid. Clearly, a lower condition number leads to a more sphere like search region and that will make the search more efficient and fast.

**Figure 2:**
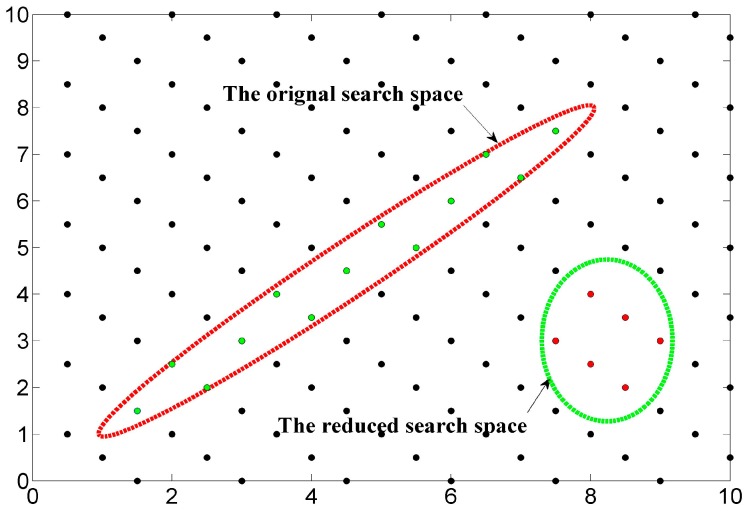
The effect of lattice reduction on the searching space.

In order to illustrate the influence of condition number on the search time, Figure 2 shows a red ellipsoid defined by the original matrix and a reduced ellipsoid. The initial value for the search is often acquired by using Babai’s method [23]. Supposing that we use the sphere search, then the minimum searching radius is the major axe of the hyper-ellipsoid. Clearly, by reducing it to the green ellipsoid showed in Figure 2, the size of the searching sphere is significantly shrunk down. Therefore, the condition number in some way reflects the search space for sphere searching method, which can be taken as an effective measure of reduction quality.

### 4.2. Experiment Design

To evaluate the performance of the reduction algorithm thoroughly, the simulation matrix B should be designed carefully. Therefore, two major parameter should be focused on in particularly: the dimension *m* and the condition number κ. We first generate matrix B with two settings and then the covariance matrix $Q_{\hat{z}}^{}$ is calculated accordingly. Note that the weight matrix W is set to I without losing generality, as it has been incorporated into the basis as $B=W^{1/2}B^{\prime }$. The two simulation settings are:

**Case 1**: The m×m coefficient matrix $B_{orignal}$ is firstly generated randomly by using the standard Gaussian normal distribution and then decomposed into matrixes U·S·V with the singular value decomposition. To control the condition number of the covariance matrix $Q_{\hat{z}}^{}$, the singular value matrix S is replaced by a diagonal matrix $S^{\prime }$, where s′1=2−κ4, s′m=2κ4 and other entries of $S^{\prime }$ are randomly distributed between ${s^{\prime }}_{1}$ and ${s^{\prime }}_{m}$. The matrix B is then reconstructed as $B=U\cdot S^{\prime }\cdot V$ and the covariance matrix is calculated as $Q_{\hat{z}}^{}=B^{T}\cdot B$ accordingly. Therefore, the condition number $\kappa_{orig}$ of $Q_{\hat{z}}$ is set to $2^{\kappa }$ with κ=5,6,7⋯16. For typical RTK application with all GNSS constellations like GPS, GLONASS, Galileo and BDS, setting the dimension of coefficient between 10 and 30 and the condition numbers between $2^{8}$ and $2^{16}$ will cover most of the situations [12].

The first case covers most of the general purpose matrixes. As we paid more attention to the GNSS carrier phase resolution application of the lattice reduction algorithm, the unique features of the real GNSS signal should be taken into consideration. In RTK applications, the integer candidates are estimated mainly by sequential integer least-squares method. As Teunissen reported in [28,29], the spectrum of conditional variances shows great discontinuity during the sequential search. And the most significant gap always shows up between the third and the forth conditional variances. 

This phenomenon can be briefly explained as follows. When solving the single short-baseline RTK positioning equations with double-difference carrier phase measurement, (*m* + 3) unknowns are involved: *m* double-difference ambiguities and a 3-dimensional baseline vector. Assuming that three out of the *m* double difference ambiguities are already resolved, the baseline vector is then solvable with the corresponding three observation equations. At this moment, the other (*m −* 3) double-difference ambiguities can also be solved precisely with (*m −* 3) remaining observation equations. The conclusion is thus reached that once 3 ambiguities (with high conditional variances) are known, the remaining ambiguities can be determined with a very high precision (which means lower conditional variances). Here we use Case 2 to generate matrixes with this discontinuity features.

**Case 2:** Let $B=U\cdot S^{\prime }\cdot V$, where U and V are obtained through the singular value decomposition like in Case 1 and $S^{\prime }$ is set to $S^{\prime }=diag\left(\sqrt{200},\sqrt{200},\sqrt{200},\sqrt{0.1}\cdots \sqrt{0.1}\right)$, mimicking this discontinuity in the spectrum of the conditional variances.

Note that Case 2 is unlike the simulation case 4 in [11]. Instead of controlling the shape of the spectrum of conditional variances (the diagonal matrix in L^T^DL decomposition) directly, Case 2 here controls the distribution of eigenvalues, which makes the generated matrixes share the same condition number.

In addition, the parameter δ is set to 0.75 for all the simulations considering of generality.

### 4.3. Performance of N-Dimensional LLL Reduction

#### 4.3.1. Reduction Quality

The condition number κ mentioned in Section 4.1 describes how the search area looks like. In order to compare the LLL reduction algorithm and the *n*-LLL reduction algorithm, we let $Q_{orig}=B_{orig}^{T}\cdot B_{orig}$, $Q_{L^{3}}=B_{L^{3}}^{T}\cdot B_{L^{3}}$ and $Q_{nL^{3}}=B_{nL^{3}}^{T}\cdot B_{nL^{3}}$ be the original covariance matrix, the LLL reduced covariance matrix and the *n*-LLL reduced covariance matrix and let $\kappa_{orig}$, $\kappa_{L^{3}}$, $\kappa_{nL^{3}}$ be the condition numbers accordingly. Therefore, the improvement of the searching area can be described as:

(42) dκo−L3=log10(κorigκL3)dκo−nL3=log10(κorigκnL3)dκL3−nL3=log10(κL3κnL3)

We run the two cases mentioned above with three variables: (i) *m*, the dimension of the original coefficient matrix from 10 to 30 with an interval of 2; (ii) $\kappa_{orig}$, the condition number of covariance matrix from 8 to 16 with an interval of 1; (iii) *n*, the parameter for *n*-LLL reduction algorithm from 2 to *m* with an interval of 2. And for each setting, we perform 1000 independent random simulations to evaluate the algorithm.

**Figure 3:**
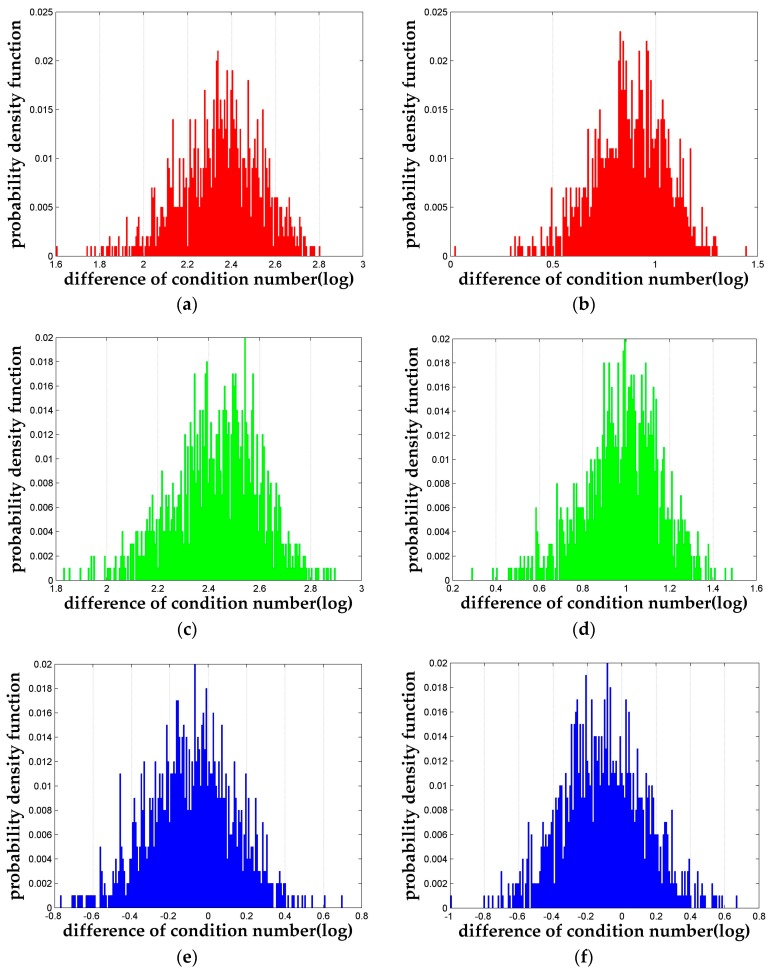
The probability density functions of the differences of condition numbers in logarithm to base 10. (**a**) *d*κ between the original matrix and LLL reduced matrix for κ_orig_ = 2^12^ of Case 1; (**b**) *d*κ between the original matrix and LLL reduced matrix for Case 2; (**c**) *d*κ between the original matrix and 4-LLL reduced matrix for κ_orig_ = 2^12^ of Case 1; (**d**) *d*κ between the original matrix and 4-LLL reduced matrix for Case 2; (**e**) *d*κ between the LLL reduced matrix and 4-LLL reduced matrix for κ_orig_ = 2^12^ of Case 1; (**f**) *d*κ between the LLL reduced matrix and 4-LLL reduced matrix for Case 2.

Firstly, we pick out a set of simulations where $\kappa_{orig}=2^{12}$(for Case 1 only), m=20 and n=4 and the probability density function of the dκ is plotted in Figure 3. It can be seen clearly in Figure 3a,b ($d\kappa_{o-L^{3}}$) and Figure 3c,d ($d\kappa_{o-nL^{3}}$) that both the LLL reduction algorithm and the *n*-LLL reduction algorithm are able to reduce the condition number significantly. Furthermore, Figure 3e,f shows the difference between the two algorithms $d\kappa_{L^{3}-nL^{3}}$, which proves the 4-LLL reduction algorithm has larger probability to perform better than the original LLL reduction. Statistically, among the 1000 independent runs of this particular simulation setting, 64.7% results of the 4-LLL is better than that of the LLL with a maximum improvement factor of 5.85. And the average improvement is about 39%. It should be pointed out that for all the simulation settings mentioned above, the conclusion showed in Figure 3 holds in general.

**Figure 4:**
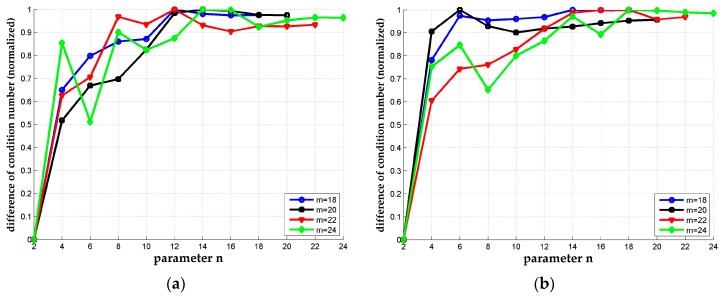
Normalized difference of condition numbers. The differences of condition number between *n*-LLL and the original LLL are normalized and plotted altogether with *m* range from 18 to 24. (**a**) The normalized difference of condition number for κ_orig_ = 2^14^ of Case 1; (**b**) The normalized difference of condition number of Case 2.

In Section 3.2, inequality (28) implies that the lower bound of the reduction quality (the orthogonality of the coefficient matrix) improves as the parameter *n* increases. To prove this conclusion, we take m=20 for example and let the parameter n vary from 4 to 20. The results shown in Figure 4 is not as expected and an upper limit shows up for the dκ as the *n* increases. And the condition number of the output matrix changes no more after *n* reaches a certain number. The explanation for this phenomenon is that the column vectors in B cannot become “more ordered” to improve the orthogonality anymore at this point. However, Figure 4 still shows that n=2 (the original LLL) is generally not this upper limit and there is space to improve reduction performance by increasing the parameter *n*.

**Figure 5:**
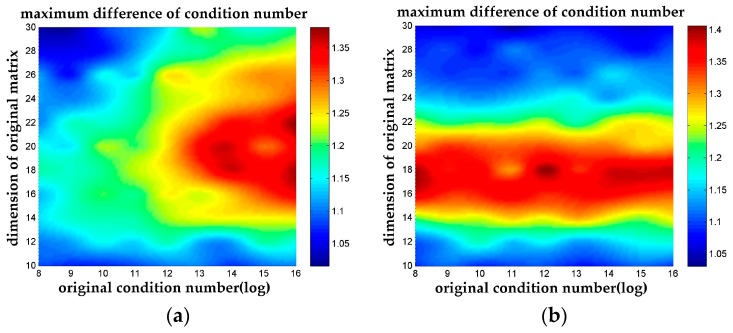
The maximum difference of condition numbers. The max value of the difference of condition number between *n*-LLL and LLL among all possible *n* parameter is plotted in the thermodynamic diagram. (**a**) The maximum difference of condition numbers for Case 1; (**b**) The maximum difference of condition numbers for Case 2.

This can be clearly seen in the thermodynamic diagram Figure 5, where the temperature denotes dκL3−nL3max=log10(κL3min(κnL3)). The highest temperature shows up when the dimension of the coefficient matrix is around 20 with a relatively high $\kappa_{orig}$ for Case 1, which indicates that the *n*-LLL reduction algorithm maximum its performance in that area. And for Case 2, the highest temperature also shows up around the dimension of 18 to 20. However, the difference between LLL and *n*-LLL becomes less significant when dealing with matrixes with higher dimension but lower condition number.

**Figure 6:**
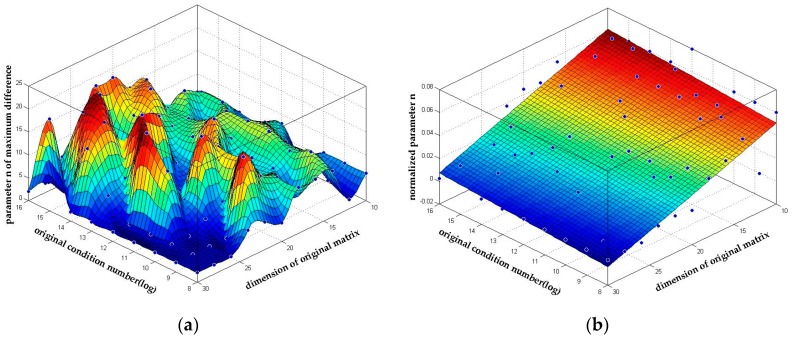
The parameter n with the best reduction quality. The corresponding parameter n of the max difference of condition numbers in Figure 5a. (**a**) The distribution of parameter n with the best reduction quality; (**b**) The 1st order fit of normalized parameter *n*/*m*^2^.

As can be seen later in Section 4.3.2, setting a higher parameter *n* always results in a longer reduction time. Thus, finding an optimized *n* to balance the performance and the reduction time is important. We analyzed the simulation results deeply and found that parameter *n* is relevant to the square of the dimension. We give out the fit result based on our simulation in Figure 6 and (43). The goodness of the first order polynomial surface fitting in Figure 6b reaches 0.6851, which means it can be taken as an empirical reference formula within the simulation setting range.

(43) $$n_{fit}=\left(0.0670-0.0027\cdot m+0.0014\cdot \log_{2}\kappa_{orig}\right)\cdot m^{2}$$

#### 4.3.2. Complexity

In order to evaluate the effect of pivoted reflection, we compared the *n*-LLL reduction without pivoted reflection (PR), the *n*-LLL reduction with pivoted reflection and the original LLL reduction algorithm. The simulation is performed on an E5-2650 CPU which makes the executing time is relatively short. Thus, matrixes with high dimension and high condition number are chosen in this simulation to obtain more accurate time measurements. 1000 independent runs are performed for 30-dimensional matrixes with condition number range from $2^{10}$ to $2^{16}$. And to evaluate the worst case of the *n*-LLL reduction algorithm, parameter *n* is fixed to 30 accordingly. Table 1 shows the simulation results, indicating that the pivoted reflection is very effective on improving the efficiency of the algorithm.

**Figure 7:**
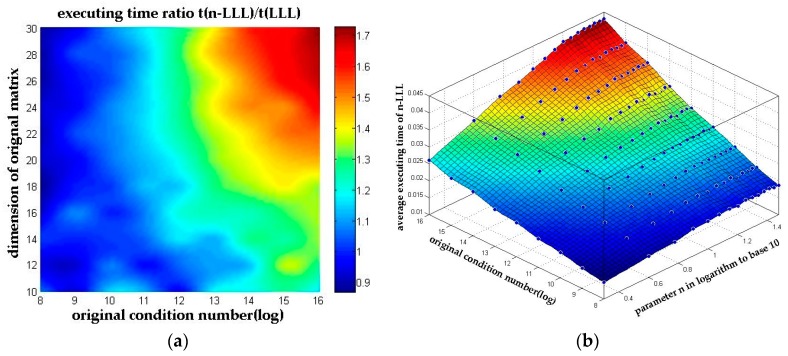
The executing time of *n*-LLL. (**a**) The executing time ratio between *n*-LLL and the original LLL algorithm; (**b**) The average executing time of *n*-LLL with parameter *n* presented in logarithm to base 10.

The conclusion is obvious that the pivoted reflection can reduce the reduction time by up to 57% and it is also clear that the *n*-LLL reduction algorithm with pivoted reflection causes no significant rise on the total reduction time compared with the original LLL reduction. And in the worst case scenario of all our simulations, the *n*-LLL reduction algorithm consumes no more than 1.72 times of the original LLL reduction time, which can also be observed in Figure 7a.

Take another look at the results in Figure 7a in a different angle where the parameter *n* is present in logarithm to base 10. The executing time in Figure 7b shows great linearity to the logarithm of parameter *n*, which meets the conclusion presented in Section 3.2.2 well. 

**Figure 8:**
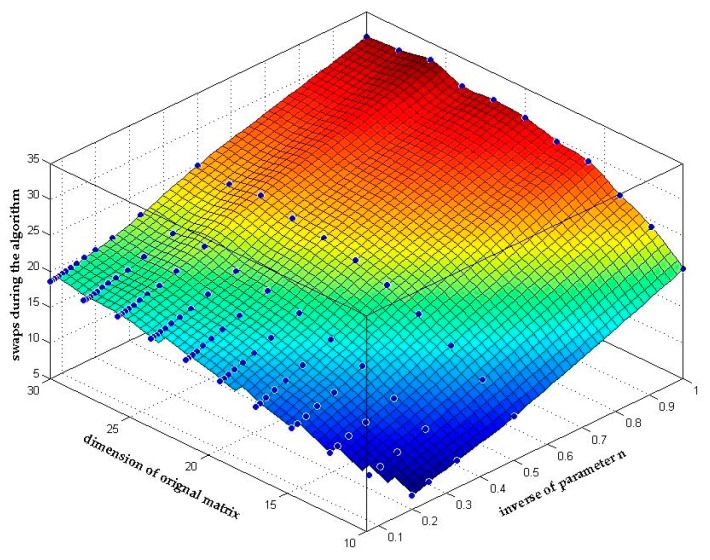
Number of swaps during the *n*-LLL algorithm.

Figure 8 shows the relationship between the matrix dimension, the inverse of parameter n and the average number of swaps in the reduction process. As we analyzed in Section 3.2.2, (39) shows that the number of swaps will actually decline with the increase of parameter *n* as long as both the dimension and the condition number of the original matrix is determined. And this conclusion can be clearly seen in Figure 8 that the number of swaps declines by *n* times.

## 5. Conclusions

This paper has presented a new kind of lattice reduction algorithm motivated by the original LLL reduction. In order to put more emphasize on the order of the basis vectors, the Lovász condition is further strengthened, expanding the 2-dimensional local optimization to n-dimensional global optimization. The pivoted reflection method based on the Householder transformation and QR decomposition is added to the algorithm in order to reduce the additional computational effort brought by the extra vector swaps. 

By utilizing the Hadamard Inequality and the orthogonality defect factor, the relation between the n parameter of *n*-LLL and the reduction quality is illustrated. Analysis shows that with the increase of the parameter *n*, the basis vectors will become more orthogonal or at least its orthogonality defect factor will have a smaller lower bound. The complexity of the *n*-LLL algorithm is estimated afterwards, which shows the *n*-LLL algorithm consumes more elementary steps than the original LLL with n>2. However, the analysis also proves that the *n*-LLL algorithm still remains a polynomial-time algorithm.

In the numerical simulation, two basic cases are covered to mimic the features of GNSS double-difference carrier-phase measurements. The simulation results show that the reduction quality of *n*-LLL algorithm is better than the original LLL reduction algorithm, especially for highly ill-conditioned matrixes. However, with the increase of the parameter *n*, a certain upper bound of the reduction quality of *n*-LLL is found during the simulation. At this certain point, the effect of permutation reaches its limit and further permutation will not significantly affect the reduction quality. In this case, a first-order surface fit is given to estimate this certain parameter *n*. 

In order to evaluate the effect of pivoted reflection, the *n*-LLL reduction with and without pivoted reflection as well as the original LLL reduction are compared. The results clearly show the power of pivoted reflection, especially for matrix with relatively low condition number. And in the worst case scenario of all our simulation, the pivoted reflection ensures the *n*-LLL reduction algorithm consumes no more than 1.72 times of the original LLL reduction executing time.

Both the analysis and the simulation results have shown that the *n*-LLL algorithm is better than the original LLL reduction algorithm, especially for highly ill-conditioned matrixes. And the increase of the total reduction time is fairly acceptable, which makes the *n*-LLL a new practical algorithm for lattice basis reduction.

## Figures and Tables

**Table 1:** The executing time of *n*-LLL reduction and LLL reduction.

Condition Number	*n*-LLL without PR	*n*-LLL with PR	Original LLL
$2^{10}$	536 ms	230 ms	217 ms
$2^{11}$	561 ms	251 ms	224 ms
$2^{12}$	595 ms	286 ms	222 ms
$2^{13}$	628 ms	316 ms	220 ms
$2^{14}$	651 ms	341 ms	222 ms
$2^{15}$	672 ms	369 ms	235 ms
$2^{16}$	714 ms	412 ms	239 ms
